# The Institutional Design of Scientific Advisory Boards on Climate Change: A Comparison at the Intergovernmental, Supranational, and National Level

**DOI:** 10.1002/gch2.202400371

**Published:** 2025-08-05

**Authors:** Helena Seibicke

**Affiliations:** ^1^ Department of Political Science University of Oslo Boks 1072 Blindern Oslo NO‐0316 Norway

**Keywords:** European Scientific Advisory Board on Climate Change (ESABCC), evidence‐informed policymaking, global climate governance, institutional design, scientific advisory bodies (SABs)

## Abstract

This article investigates the institutional design of scientific advisory bodies (SABs) on climate change across three levels of governance: intergovernmental Intergovernmental Panel on Climate Change (IPCC), national (UK's Climate Change Committee (CCC)), and supranational (European Scientific Advisory Board on Climate Change – ESABCC). Drawing on original empirical data and comparative analysis, the paper examines how institutional features (mandate, composition, autonomy, capacity, and knowledge provision) shape the potential roles and influence of these bodies in climate governance. The ESABCC, established in 2021, represents a novel institutional innovation within the EU's climate policy architecture. As the first supranational advisory body of its kind, it navigates a complex political space, balancing scientific independence with embeddedness in European Union's (EU) policymaking structures. Through a comparative lens, the analysis shows that while all three bodies aim to provide credible scientific input, their design reflects different governance logics and degrees of proximity to policy. The paper argues that institutional design is a critical determinant of how effectively SABs contribute to legitimate, evidence‐informed climate policy. By mapping the ESABCC's position within the EU's multi‐level governance framework, the study highlights its evolving role and outlines the implications for the broader use of expert knowledge in turbulent policy environments.

## Graphical abstract

This article compares the institutional design of the Intergovernmental Panel on Climate Change (IPCC), UK's Climate Change Committee, and the European Scientific Advisory Board on Climate Change (ESABCC). It reveals how the institutional design at different governance levels shapes advisory bodies' roles and influence in climate policymaking, highlighting the ESABCC as a novel supranational actor navigating science‐policy boundaries in the EU's evolving climate governance architecture.

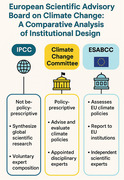

## 1 Introduction

Globally, science advisory bodies exist or are being established on multiple governmental levels (national, regional, municipal) to inject science into policymaking processes and enhance governmental accountability. As part of this increasing trend, yet unique in its position at the supranational level, the European Scientific Advisory Board on Climate Change (ESABCC)^[^ ^1^ ^]^ was established in 2021 under the European Climate Law as a novel independent body which provides scientific knowledge to the European Union's (EU) institutions. This article asks how the institutional design features of the ESABCC shape its potential role in the EU's wider climate governance framework. To this end, I draw on several theoretical strands of scholarship, ranging from public administration to STS, as well as original empirical data. I investigate to what extent the institutional design of the ESABCC is an innovation at the supranational level in comparison with climate advisory bodies at the intergovernmental and national level. To glean a better understanding of the ESABCC's institutional set‐up, internal processes and nature of science advice (i.e., knowledge basis and production processes), I analyze the Board in comparison to the Intergovernmental Panel on Climate Change (IPCC) and the British Climate Change Committee (CCC). This choice is based on a) the importance of the IPCC reports in the guidance and formulation of EU climate targets, and b) the CCC's status as the first climate advisory council of its kind, serving as a blueprint for the institutional design of other climate advisory bodies. Moreover, with the ESABCC being situated at the supranational level, it uniquely sits between existing advisory bodies at the intergovernmental (i.e., the IPCC) and national (i.e., the CCC) level.

Despite the growing literature on scientific advice aimed at tackling the causes and effects of climate change, there is a sparsity of in‐depth studies concerned with the institutional design (i.e., internal structures and processes) of scientific advisory bodies (SABs) (see notable exceptions^[^ ^2^, ^3^, ^4^ ^]^). This is surprising as policymakers and scholars alike have increasingly emphasized the importance of evidence‐informed decision‐making in climate policy.^[^ ^5^, ^6^ ^]^ For decisions to be genuinely guided by scientific evidence, policymakers routinely seek out this knowledge and SABs are often a critical part of this process.^[^ ^7^ ^]^ Climate advisory councils can thus potentially shape climate policies and can have political influence through the production of climate knowledge, advice, and evaluation processes.^[^ ^8^ ^]^

The article contributes to existing scholarship which stresses the importance of the institutional design of advisory bodies to successfully perform their functions.^[^ ^9^, ^10^ ^]^ Valuable insights can be derived from studying the institutional design of SABs, not only regarding the functioning of these institutions, but also to better understand how knowledge for (environmental) governance is enacted in practice.^[^ ^11^ ^]^ After three decades of climate action, the focus increasingly has shifted to the socio‐economic impacts of policies, as it is “the politics of who does what, where, when and in what order”^[^ ^12^ ^]^ that is holding society back from effectively addressing the climate emergency. Concomitantly, what is needed is a better understanding of “who does climate governance”.^[^ ^13^ ^]^ As SABs provide expertise and knowledge which shape climate policies, they can have far‐reaching (political) influence over their substance and direction.

Moreover, focusing on the institutional design of SABs is crucial for several reasons: Well‐designed advisory bodies enhance the credibility and trust of the scientific advice they provide.^[^ ^14^ ^]^ This is especially important in complex and contentious policy areas such as climate change.^[^ ^15^ ^]^ Independent and robustly designed advisory bodies are more likely safeguarded from politicization and undue influence (Wright 2024). Institutionalized advisory structures with clear mandates also facilitate accountability, efficiency, and enable better policy coordination with other institutions, stakeholders, and other scientific actors.^[^ ^16^ ^]^ This then results in increased policy relevance, ensuring that the advice is not only scientifically sound, but also “actionable” and impactful.

Last, proper institutional design enhances the legitimacy of scientific bodies themselves. This is important in gaining acceptance and support for scientific advice, in turn, influencing the aforementioned overall trust in scientific advice. Thus, the institutional design is not just an administrative concern but a foundational element that influences the effectiveness, impact, and trustworthiness of SABs.

This paper systematically compares the institutional design features of the IPCC and the CCC with the ESABCC. Looking at internal institutional design helps when assessing what type of advisory body the ESABCC is (and can be), its role in the EU's climate governance, its relationship vis‐à‐vis other stakeholders and institutional actors, contributing to an emerging body of research examining climate governance frameworks and institutions,^[^ ^17^, ^18^, ^19^ ^]^ in particular on the pivotal role played by independent expert advisory bodies.^[^ ^20^, ^21^ ^]^

The article is organized as follows: Section two and three delves into the existing literature on the constitutive framework of SABs (climate framework laws (CFL) and institutional design) and on scientific advice on multiple levels. Section 4 provides an overview of the methodology and data material. The theoretical and empirical insights pave the way for a systematic and comprehensive comparative analysis of intergovernmental, national, and supranational SABs in Section 5. This is followed by a concluding discussion reflecting on the importance of the ESABCC's institutional design for its evolving role and outlines the implications for the broader use of expert knowledge in turbulent policy environments in Section 6.

## 2 Constitutive Framework of Climate Advisory Bodies

The analysis in this article draws on several existing bodies of literature in order to understand how the organizational structure of the ESABCC can shape its role in the EU's climate governance framework. First, the extensive literature on the institutional design features of EU agencies; second, scholarship on SABs; and third, multiple perspectives on scientific advice at the policy‐science nexus.

CFL are designed to provide legally binding, long‐term policy frameworks for climate action. While some provide a legal foundation for governmental processes committed to carrying out specific measures under the United Nations Framework Convention on Climate Change, others set out a framework of laws, objectives, or general procedures for climate policy developments.^[^ ^22^ ^]^ A defining trend which can be observed in CFL is the inclusion of SABs, tasked with monitoring progress toward climate targets, providing scientific input to policymaking, and holding governments accountable (^[^ ^11^ ^]^ Setzer and Higham 2022).^[^ ^23^, ^24^ ^]^ Most CFLs mandate SABs to provide independent scientific advice, thereby enhancing the input, throughput, and output legitimacy of EU climate policies. I will return to the specific legal foundations of the supranational, national, and intergovernmental cases of SABs studied in this paper in Section 6.

### 2.1 The Importance of Institutional Design

The design features of bureaucratic bodies matter for how they function in terms of policy influence, independence, expertise/ knowledge use, and accountability mechanisms. Moreover, the institutional design shapes the balance between technocratic authority and political influence.^[^ ^25^ ^]^ For bodies whose authority rests on scientific/ technical expertise and knowledge provision, such as regulatory agencies and advisory bodies, organizational design features determine the procedural transparency, stakeholder inclusion, knowledge representation, and openness to scrutiny.^[^ ^14^, ^26^ ^]^ Moreover, the bodies’ mandate shapes their role vis‐à‐vis other institutional actors,^[^ ^27^ ^]^ institutional linkages in multi‐level systems (Skjærseth 2021), organizational reputation,^[^ ^28^, ^29^, ^30^ ^]^ and knowledge uptake.^[^ ^31^ ^]^

The literatures on EU agencies and SABs overlap in significant conceptual and empirical ways, as both are understood as non‐majoritarian institutions (Majone 1996) that operate at the policy‐science nexus, which informs decision‐making through their technical, scientific, or specialized knowledge. While both types of institutions depend heavily on their technocratic acumen (for EU agencies through effective regulation and technical competence; for SABs through epistemic trustworthiness^[^ ^14^ ^]^), institutional design features, such as formal independence, appointment processes, budgetary autonomy, and transparency, are also important key variables to explain their effectiveness, overall scientific and democratic quality of policymaking or the role they play in and the influence they have on political systems.

Recent literature on SABs has offered critical insights into variables such as contextual background, mandate, structure, or process of issuing advice.^[^ ^3^, ^32^ ^]^ Advisory bodies can have several goals and objectives; however, there is a scarcity of work providing critical comparative analysis of their institutional design features, practices, and objectives.^[^ ^33^ ^]^ The following section details the methods used for the empirical work for this article, in order to address this significant research gap before offering a comparative analysis of cases of SABs at the supranational, national, and intergovernmental level.

## 3 Scientific Advice

In today's global context, interactions at the policy‐science nexus are often fraught with friction. This is not because scientific experts are somehow less relevant or less needed in democratic politics; on the contrary. Experts are needed more than ever, yet experts enjoy less credibility and public trust than ever–a state of affairs Weingart^[^ ^12^ ^]^ refers to as the “paradox of expertise.”^[^ ^34^ ^]^ Regarding climate policymaking, scientific experts are called upon to increase their feasibility, and public acceptance, as well as their democratic and scientific legitimacy. Scientific expert bodies, such as climate advisory councils, represent concrete policy innovations at multiple levels of governance. The global emergence and proliferation of these councils indicate the expansion of national climate institutions whose role is to produce climate knowledge and advice on and evaluate climate policies.^[^ ^8^ ^]^

Jasanoff^[^ ^35^ ^]^ argues that offering “serviceable truths” is fundamental to the professional domain of science advising. In this sense, the output of SABs differs from academic science; it is their expertise that informs regulation and policymaking through the generation, synthesis, transformation, and assessment of knowledge related to specific policy problems (^[^ ^36^ ^]^ 354).

Scientific advice constitutes a separate professional domain, governed by particular standards, norms, and institutional rules, functioning as an intermediary organizational form located at the policy‐science nexus. Furthermore, according to Lentsch and Weingart, such advisory bodies are characterized by their institutionalization, being statutory entities, performing their operations independently of external influences, particularly from their clients, basing their activities on scientific expertise, and primarily tasked with advising the legislative or executive branches, such as the government or government departments, on science‐related policy issues.

### 3.1 Multilevel Scientific Advice

#### 3.1.1 The Intergovernmental Level

SABs at the intergovernmental have played a crucial role in informing and shaping policies across a range of global issues, such as climate change and public health. Prominent examples are the IPCC, the Intergovernmental Science‐Policy Platform on Biodiversity and Ecosystem Services, and the World Health Organization's advisory committees. Intergovernmental science advisory bodies are characterized by several key institutional design features that are aimed at enhancing their ability to provide credible and policy‐relevant scientific advice. These bodies operate with a clear mandate often defined by international agreements, aiming to deliver policy‐relevant but not policy‐prescriptive guidance. They are structured to include representatives from multiple countries (i.e., intergovernmental), gathering input and legitimacy through plenary sessions. Smaller governing bodies, such as bureaus or secretariats, are responsible for the administrative work.

The inclusion of multidisciplinary expert panels ensures comprehensive assessments, with sub‐groups focusing on specific themes or aspects of their overarching mandates. The assessment and reporting processes are rigorous, involving periodic reports that synthesize the latest scientific knowledge and undergo extensive peer reviews to ensure accuracy and credibility. Transparency and inclusiveness are central to their operations, with open processes and mechanisms for stakeholder engagement from various sectors, including NGOs, industry, and civil society. These bodies also prioritize capacity building through training, workshops, and knowledge transfer initiatives, especially in developing countries. Funding is typically derived from multilateral sources, maintaining operational independence, while dedicated communication strategies ensure that findings are effectively disseminated to diverse audiences. Continuous evaluation and feedback mechanisms are in place to monitor and improve their processes and outputs, ensuring ongoing relevance and effectiveness in bridging the gap between science and policy at the global level.

#### 3.1.2 The National Level

While science advice at the supranational level has done a substantial part in putting climate change on the political agenda, it has increasingly become apparent that, in addition to intergovernmental action, climate change policies critically depend on the implementation at the national level.^[^ ^37^ ^]^ Many countries have implemented national climate policies to accomplish pledged Nationally Determined Contributions under the Paris Climate Agreement. National science advisory bodies play a crucial role in shaping how these targets are implemented by providing scientific knowledge and evidence‐based recommendations to national governments. By issuing annual progress reports, formulating (in some cases, legally binding) carbon budgets, or evaluating and monitoring governments’ performance, these bodies potentially contribute to the effectiveness and legitimacy of climate governance processes.

Comparative studies of these advisory bodies reveal a variety of organizational structures and operational methods that reflect the diverse political and administrative cultures in Europe.^[^ ^38^, ^39^ ^]^ In addition to the varying political contexts and climate governance systems across countries, SABs can be characterized across different dimensions. After briefly describing the methods used for the empirical analysis, the analytical framework, and the comparative dimensions for the analysis between the IPCC, CCC, and ESABCC will be elaborated on in more detail.

## 4 Method

This study employs a multi‐method approach to investigate the role and functions of the ESABCC within the EU climate governance framework. The methods include document analysis, interviews, and multimedia analysis.

To understand the policy and legislative context in which SABs operate, I conducted a comprehensive document analysis. This involved the systematic review of relevant policy documents, legislative texts, and official reports. These documents were sourced from EU institutions, including the European Environment Agency (EEA), the European Commission (COM), and the European Parliament (EP). The analysis focused on identifying key themes, policy directives, and legislative mandates that shape the functioning and impact of SABs in climate governance.

Additionally, to gain in‐depth insights into the practical workings and perspectives of the Board, I conducted semi‐structured interviews with key informants. While all 15 scientific experts were repeatedly contacted, only two agreed to an interview. Additionally, a key member of the ESABCC secretariat and a member of the EEA connected to the Board's work were also interviewed. The interviews were transcribed and analyzed according to the analytical framework detailed in Table S1 (Supporting Information).

To complement the document and interview data, I conducted a multimedia analysis, examining both audio and visual content.

The data material included podcasts, webinars, and audiovisual recordings (for a detailed list, see Table S2, Supporting Information). By triangulating data from document analysis, interviews, and multimedia analysis, this study provides a robust and comprehensive understanding of the roles and functions of the ESABCC within the EU climate governance framework.

## 5 Multilevel Climate Science Advice: Comparing Intergovernmental, National, and Supranational Advisory Bodies

In this section, the IPCC, CCC, and ESABCC will be discussed in terms of the following analytical framework: mandate, composition, capacity, focus, role, type of knowledge provision, and degree of separation. These dimensions are drawn from existing studies of (climate) advisory bodies, such as Acosta et al.,^[^ ^33^ ^]^ Averchenkova and Finnegan (2021), De Donà and Linke,^[^ ^10^ ^]^ Hoffman et al.,^[^ ^7^ ^]^ etc.,^[^ ^40^, ^41^, ^42^ ^]^

In the following comparative analysis, a brief overview of the IPCC (situated at the intergovernmental level) and the CCC (at the national level) are given, while the ESABCC–situated between the two levels–will be described in greater detail.

### 5.1 The IPCC (Supranational)

The IPCC, established in 1988, aims to provide scientific guidance on the magnitude and temporal progression, impact, and responses of climate change. It is loosely composed of thousands of scientists involved in drafting reports, which then require approval from the governments of the 195 member states. The IPCC's secretariat consists of 10–12 staff members, with the Bureau consisting of 32 expert members.

Focusing on physical science, adaptation, and mitigation, the IPCC's role is advisory. It does not produce original science, but rather reviews and synthesizes the latest climate research in an effort to build consensus among both scientists and policymakers. Its reports aim to be “neutral, policy‐relevant but not policy‐prescriptive” (IPCC). As a boundary organization, the IPCC aims to enhance the cooperation between scientists and policymakers while seeking to maintain distinct boundary between both worlds.^[^ ^43^ ^]^

Operating across science, policy, and public discourse, the IPCC faces challenges avoiding the overpoliticization of science and the over‐scientization of politics.^[^ ^26^ ^]^ This has resulted in the criticism that its reports run the risk of “scientific reductionism” and emphasizing the lowest common denominator.^[^ ^40^ ^]^ Another criticism leveled against the IPCC is its stated strategy of seeking to remain policy‐relevant but not policy prescriptive.^[^ ^44^ ^]^ Without setting out specific sets of policy instruments and concrete actionable policy measures, the bodies’ relevance has been called into question.^[^ ^13^, ^45^ ^]^

Key challenges also include the close supervision by interstate hierarchies, the political challenge of communicating scientific uncertainty, misinformation campaigns by powerful vested interests, and legitimacy deficits.^[^ ^22^ ^]^ Some have concluded that the IPCC's set up as an intergovernmental organization is fundamentally flawed, so that “the degree to which the IPCC is capable of generating usable knowledge is largely politically circumscribed” (^[^ ^46^ ^]^: 145). There is a desire to speak with a single voice, through strong focus on consensus. Criticized the overly close relationship between scientists and policymakers, as the former adapting to what they consider politically possible for the latter to digest.^[^ ^40^ ^]^ Through the organization of the work cycles into consecutive phases,^[^ ^47^ ^]^ the relationship between the IPCC's and the policymakers can be characterized as both integrated and separated.

### 5.2 The UK's CCC (National Level)

The passing of the UK's Climate Change Act in 2008 has widely been heralded as a significant event in global climate governance.^[^ ^48^ ^]^ The legislation stipulated, amongst other innovations, the establishment of an independent expert panel. The CCC's institutional set‐up is widely viewed as a good practice example, and it has provided inspiration for a many of the recently established bodies in the EU (i.e., ESABCC) and beyond. Its mandate encompasses tasks such as the provision of independent advice on climate change adaptation and mitigation; monitoring progress on reducing emissions and enhancing climate resilience; conducting independent analysis into climate science; engaging with wide range of stakeholders to share evidence and analysis (CCC).

At the time of writing, the CCC is composed of 12 members in total. With the exception of the chair, who is a high‐profile politician, most members are technical experts who are chosen and appointed by national authorities on the basis of their specialized knowledge and experience. Each member has a fixed term appointment of five years, with the possibility of reappointment (^[^ ^22^ ^]^ 64). The scientific experts are supported by a sizeable secretariat of 50 analytical full time equiv. staff working across mitigation and adaption, as well as eight corporate support staff (CCC). The Committee has two subcommittees, one on mitigation and one adaptation, which is reflected in the focus of its outputs.

The CCC publishes annual reports aimed at scrutinizing the UK governments’ policies to which the government is obliged to respond in a report to parliament. Each annual report contains the CCC's assessment of existing policies, as well as recommendations about how to address any gaps.^[^ ^21^ ^]^ This, in turn, creates a system of accountability where the government must provide a formal written explanation any time it deviates from the CCC's recommendations.^[^ ^2^ ^]^

Regarding the issue of separation of integration, the Committee has faced several allegations of conflict of interest and its governance arrangements with sponsoring departments are criticized for being outdated. Through expanding its remit and adopting an active public profile, critics argue further that its independence is undermined, and it has become a political actor too far integrated in the politicized processes of the UK government, rather than delivering balanced scientific advice (see for example^[^ ^49^ ^]^).

### 5.3 ESABCC (Supranational Level)

This section provides an in‐depth discussion of the ESABCC's institutional design, operationalizing the analytical framework with the original data gathered through the expert interviews, multimedia and document analysis. Through the empirical work, unique insights on the internal processes of the ESABCC have been gained. Moreover, through the empirical analysis, we also gain a better understanding of how the institutional design of the ESABCC is implemented in practice. The overarching categories are broken down into more detailed subpoints.

#### 5.3.1 Mandate

The mandate of the ESABCC is rather broad. Its core task is to “provide scientific advice and issue reports on existing and proposed Union measures, climate targets and indicative green‐house gas budgets” (Art. 3.2.(b)). The Advisory Board was established through an amendment of the founding regulation of the EEA in 2021. It delivers concrete advice, recommendations, and guidance to EU institutions (European Commission, EP and Member States), standing on facts, the best available and most recent scientific evidence, and robust analysis. To ensure its inputs are relevant and timely, the Advisory Board maintains a regular dialogue with policy makers at the EU level and seeks to engage early on in EU policy processes (ESABCC 2023).

The EP successfully proposed for the Board's establishment. However, the establishment of the Board faced some resistance. To some policymakers it seemed undesirable to add an additional advisory body to the already complex policymaking process in Brussels. The interviewees pointed out that Commission was not initially supportive of the Board's creation. In the past, there already had been a strained relationship between the Commission (especially DG Environment) and the EEA, and the Board's establishment added to the institutional power dynamics within the Commission and the EEA. With the Board's establishment, DG ENVI faced losing key responsibilities for implementing the EU's climate policies to DG CLIMA, which has been tasked to deliver the European Green Deal.

The EEA, apart from some exchanges with the different institutions regarding some design elements, had no role in the institutional design of the ESABCC. The EEA's perspective emphasized that “there would not be a new body that would do duplicate what the agency (EEA) was already doing, because the agency, to some extent does a bit of what some national councils do, which is to take stock on progress on a regular basis, a bit like also the European Commission does” (ESABCC 3).

An important consideration in the establishment of the ESABCC was its institutional design in relation to the IPCC. As one of the interviewees pointed out, one consideration was “to avoid having something that looks like a European or regional, regional IPCC that could open Pandora's box basically, where if you have a European IPCC, you could have an American IPCC, Brazilian IPCC, etc. And that would undermine the IPCC itself and undermine planet science altogether. So really, the lawmakers had to make it clear that the Board builds on the IPCC and synthesizes the best available science in a way that is relevant for EU climate policymakers” (ESABCC 3).

When asked why the Board was needed, given the many available sources of scientific evidence related to climate change, it was pointed out that the climate law is the formal justification for the Board. However, it was also emphasized that the Board does not want to duplicate:

“It is important to understand that we are an independent, scientific reference point; but we have to take into account a very specific decision‐making framework. We provide the scientific knowledge for the EU to reach the climate neutrality goals and the pathways should be underpinned by scientific expertise and up‐to‐date information. Of course, you can ask the question, why should policymakers not just look at the IPCC reports, and the answer is very simple: The IPCC reports are not focusing on the specifics of climate neutrality within Europe. We are basically advising the Commission and evaluate the carbon budget, but also the instruments. We are independent, we are not within the executive power of the Commission, we are on the outside, we are purely scientific. In that sense, we are not replicating or duplicating what others are doing. This is how I would describe our mandate.” (ESABCC 4).

When the Board was established, the expert from the EEA's scientific committee viewed it as a positive development. They believed that it would bring a significant increase in scientific knowledge and rigor to EU policymaking. The direct line of influence between the climate board and EU policymakers was seen as a crucial aspect. Moreover, the fact that it was mandated by law for European policymakers and the European commission to seriously consider the Board's advice was seen as a major improvement, since “we went through a period where it was hard work to get EU policymakers to listen to climate and environmental knowledge.” (EEA).

In terms of the priorities and interpretation of the mandate, the processes seem less formalized and structured than in some national advisory bodies. While the first reports were still somewhat mandated by the EU Climate Law, the Board seems to be relatively free to set its own work program and chose its own priorities (subject to the EU's policy agenda and current policy landscape): “Much of the work we have done so far was requested by the Commission or the Parliament. So, if in the procedure there is a place for us to have a voice, then of course, we need to think about whether we want to speak to this issue or not. And then we decide on whether to start working on the advice. E.g., the recommendations for the 2040 recommendations and the climate budget, it was part of the Climate Law that we should give our input to that process” (ESABCC 2).

As opposed to some national bodies, there are no structured mechanisms such as sector dialogs with legislative bodies (i.e., DGs) in charge of policymaking and implementation, as for example the Irish Climate Advisory body has with government ministries. It also is different to national advisory bodies, like the CCC, in the way that the ESABCC is not statutorily obliged to publish regular progress reports assessing the advancement of, e.g., emission reductions.

The ESABCC's annual work program sets out its long‐term priorities but also gives room for reacting to the policy context (such as the energy crisis or the war in Ukraine). One report (that one of the scientists) initiated was on the Ukraine crisis which was just an emerging issue at the time (ESABCC 1). Priorities are set via an internal call for proposals, where the scientific experts give input for the coming work program. This means there is room for the scientists to give ideas and impulses, however, the question of capacity restricts the number of tasks and topics being taken on: “We all come with suggestions… [Some things from the previous year] were delayed. So they spill over into [the next year] … [We need to be] careful not to take on too much work… Of course, everything is in consensus… Lots of agreement, different perspectives, but also respect for the different perspectives” (ESABCC 2).

#### 5.3.2 Expert Characteristics and Recruitment Procedures

The ESABCC is composed of 15 senior scientific experts covering a broad range of relevant disciplines. No more than two members of the Board shall hold the nationality of the same member state. The independence of the members of the Board shall be beyond doubt (EU Climate Law, Art. 10a). The Management Board designates members for a term of four years, which can be renewed once, following an open, fair, and transparent selection procedure. The Board is to possess varied disciplinary and sectoral expertise, as well as gender and geographical balance (ibid). The chairperson is elected from among its members for a period of four years. The Board is dominated by scientific experts with a disciplinary background in the natural sciences. It could be thus argued that it is reproducing if not strengthening a dominance of natural science in climate governance (thereby possibly limiting the type of knowledge provided) resulting in a similar reductionism that the IPCC has been criticized for.

Opposed to other advisory bodies at the national level, the Board is solely composed of scientists with an expertise in climate science. There are no public officials, civil servants, union representatives, civil society representatives, or indeed experts with a non‐climate science background. Most ESABCC experts have a background in climate‐related natural sciences (such as engineering or physics), as well as experience from involvement in the IPCC or national climate advisory boards. Indeed, in the call for scientific experts the evaluation criteria were: Scientific excellence; experience in carrying out scientific assessments and providing scientific advice in the fields of expertise; broad expertise in the field of climate and environment sciences or other scientific fields relevant for the achievement of the EU´s climate objectives; professional experience in an inter‐disciplinary environment in an international context (ESABCC 2021). The call was framed to appeal to scientists with the headline “The EU needs you! Are you an expert in a field relevant to climate change? Are you concerned that EU policies don't match the science? Do you want to push for reforms based on data not politics?” (EEA 2021).

As one expert summarizes: “With the IPCC, I think most of us, if not all of us, were a part or are part of the IPCC. Similarly, for national [advisory boards], a number of people are part of national climate boards to the degree they exist” (ESABCC1).

Experts were selected by a committee through an “open, fair, and transparent selection procedure” (JPI Climate 2022). The EEA management board was tasked with designating the members of the Board. Specifically, the evaluation committee consisted of seven members, including the Chair of the EEA Management Board, the EEA Executive Director, the Chair of the EEA Scientific Committee, and four other members of the EEA Scientific Committee. The evaluation Committee was be chaired by the EEA Executive Director. The selection committee's composition followed this design, rather than having representatives of the EU institutions present, in order to avoid political nominations. When it came to the hiring of the climate board members, members of the EEA's scientific committee were also involved in the process. As one interviewee pointed out, as the mandate of the ESABCC members is limited to a certain amount of time, it is expected that the scientific committee will be involved in the next round of membership selection (ESABCC 3).

At the time of writing, even though this is a stated goal of the Board, a geographical and gender balance among the experts has not been achieved. This is to say, the experts are predominantly male and originate from or are based in North‐Western Europe. It was mentioned by one of the interviewees, that the EEA's management board has also recognized the need to ensure greater diversity and representativeness in the composition of the Board. “There was some disappointment within the agency and the management board regarding the lack of candidates from different geographical parts of Europe in the previous selection process” (EEA). As a result, discussions are already underway to explore how to improve diversity and representation in future selection rounds.

#### 5.3.3 Recruitment of Secretariat

The Secretariat is hosted by the EEA based in Copenhagen, Denmark. It consists of eight staff members (at the time of writing, with an ongoing recruitment process towards 14 staff members), a head of secretariat. The team has interdisciplinary competencies relating to the overall mandate of the Board, covering areas such as energy, emissions trading, agriculture, transport, and technology. Besides the topical roles, there is also one communication officer and office assistant supporting the work of the secretariat.

Similar to the scientific experts, the recruitment of secretariat staff is handled through the EEA. Vacancies are published on the EEA website and staff are selected through a designated selection committee consisting of the ESABCC head of secretariat, and three EEA members. Since the secretariat does most of the report drafting, staff with knowledge on “climate policies, EU climate policies, economics, climate modeling, specific sector aspects relevant to specific sectors or to specific policy instruments” (ESABCC expert 3) were selected. In terms of disciplinary background, similar to the scientific experts, secretariat members have disciplinary backgrounds mainly in natural sciences, sector specific expertise, and economics. Reflecting the economic underpinnings of EU climate governance, “economics are really important when you want to understand policies, but also policy development” (ESABCC 3).

#### 5.3.4 Division of Labor

The working mode within the advisory board has evolved from an ad hoc to a more stable framework, with the secretariat primarily handling the drafting process under the guidance of scientific experts. The scientists’ input is particularly influential when it comes to defining the structure and messaging of the reports, as well as providing recommendations on evidence and scientific papers to be considered. The secretariat is responsible for collecting data and evidence, and then using this information as a basis for the drafting process. It has been noted by interviewees that a significant portion of the drafting work is carried out by the secretariat, while the Board plays a vital role in setting the direction and reviewing the overall quality of the work.

Additionally, in preparation of the 2040 report, the secretariat was heavily involved, collaborating extensively with IISA to access relevant databases. While the secretariat undertook the majority of the legwork, input from all members was crucial, particularly when it came to formulating the key messages, recommendations, and executive summaries. This process involved detailed discussions on how to frame and articulate the content. “I think now we are in a working mode that is probably a bit more stable, where the secretariat does most of the drafting, under the steer of the advisory board members when it comes to the structure, or when it comes to the messaging.” Or we also get input in terms of evidence and scientific papers that we should look into. And we do the groundwork, which is collect the data and the evidence, draft on that basis. But there's a strong role for the advisory board to set the direction and to review the quality of the work (ESABCC 3). Overall, the scientific experts give input on “how to frame it, how to formulate it, what to say” (ESABCC 2).

#### 5.3.5 Question of Capacity and Prioritization Process

The activities of the ESABCC are funded through the EEA budget, which is part of the overall EU budget. This is proposed annually by the European Commission and must be approved by the Council of the EU and the EP. The annual EU budget must remain within the ceilings of the Multiannual Financial Framework.^[^ ^14^ ^]^ This means that there is little capacity to fund additional work, such as research and outreach to the broader scientific community.

Due time pressures for both board members and the secretariat, there is a constant need for prioritization of tasks and projects. The time available is always deemed insufficient to cover the full spectrum of everyone's perspectives and visions. As a result, there is a continuous exercise in prioritization, focusing on important areas that have the potential to influence policy‐making processes. The scientists are expected to allocate 20% of their time to the Board's activities, creating an additional commitment on top of their existing responsibilities, which further exacerbates the issue of the Board's capacity.

Reflecting on past experiences, it has been acknowledged by several interviewees that the Board may have been overly ambitious in its endeavors, leading to significant work overload for the Secretariat. Moreover, the head of the secretariat closely monitors the policy developments in Brussels to determine the appropriate timing for publishing reports and recommendations. Delays in publishing certain reports have occurred due to the overwhelming workload and the need to consider the timing in relation to key events such as the COP. Additionally, there has been a recognition that the volume of work has been taxing for both the secretariat and the board members, making it challenging to attend multiple meetings simultaneously. Consequently, efforts are being made to adopt a more linear approach, moving from one major project to another. Overall, there is a shared understanding among members that they “cannot cover all the ground” (ESABCC 3) and therefore a prioritization is required to effectively manage time and resources.

#### 5.3.6 Focus of Climate Action Measures

Interviewees indicated that while there is a growing clarity on the need for more work on adaptation, the current thematic emphasis of policies largely remains on mitigation efforts. This direction is anticipated to persist in the near future, given that the ESABCC's forthcoming major reports will center on carbon removals and subsequently on agriculture, both of which have significant implications for the impact of climate change and potential links to adaptation. The interviewees further expressed a belief that mitigation will continue to hold strong importance, supported by the current mandate and emphasis within the EU Climate Law. Some also highlighted the need to examine specific mitigation measures that may not currently be incorporated in integrated assessment models, such as material efficiency and circular economy strategies.

#### 5.3.7 Type of Knowledge Provision

The knowledge provided by the ESABCC to EU policymakers is interpreted by the Board to fulfil a multifaceted role. Here, the focus lies on the scientific knowledge being translated for policymakers into something that is policy‐relevant, actionable, robust, and thorough. As pointed out by one of the interviewees, the recommendations of the board aim to be “policy relevant […] And it tries to go fairly deep into each of these issues. So we cannot take the risk of being superficial and come up with something that will be contested” (ESABCC 3). Another interviewee, when asked how he saw the role of the Board's knowledge provision expressed that “I understand the role is like a link between the global science and then making it actually actionable advice” (ESABCC 1). This sentiment is echoed by another respondent, who also touched upon the robustness of knowledge: “But we did not want to come up with an opinion or just a few pages; [the evidence needs to be] actionable and understandable by policymakers” (ESABCC 2).

#### 5.3.8 Key Function vis‐à‐vis Policymakers

Previous scholarship has chartered the extensive knowledge architecture needed to achieve sustainability transitions.^[^ ^23^, ^50^, ^51^, ^52^ ^]^ Scientific knowledge around the issue of climate change has been a central element in the EU's climate policy development. In line with a broader push toward evidence‐based policymaking, an extensive “knowledge exchange architecture” ‐ referring to the ways and means by which multidisciplinary scientific knowledge enters the policymaking process – has emerged (Dupont et al. 2020). With the inception of the ESABCC, a new player has entered into this framework, an interesting development given the politized nature and political salience of climate change. Moreover, with so much scientific knowledge and evidence available, it raises the question of the Board's added value in this complex knowledge exchange architecture.

One of the respondents pointed out that according to the 2040 recommendation, the Board aimed to demonstrate that it could provide scientific perspectives that were not confined to academia. Rather, these perspectives could be effectively incorporated by policymakers in shaping future targets and policies (ESABCC 3). The establishment of the European Advisory Board was driven by the EP, seeking to include an additional element of scientific input in the work of the European Commission and member states. The intention was to introduce a new player within the triangle of institutions to maintain high environmental ambition, despite challenging times (ESABCC 3).

According to another expert, the role of the IPCC primarily involves summarizing scientific evidence through reports issued every six or seven years, which serve as a stocktake of the current state and the necessary actions to achieve the desired outcomes. These reports adopt a global and generic perspective, unable to consider specific circumstances, given the existence of a single legal framework. On the other hand, the Board works continuously and provides tailored advice to policymakers when required for specific issues they are dealing with at any given time. This makes the Board's involvement more hands‐on and targeted (ESABCC 1).

Although the long‐term impact of the Board is yet to be evaluated, the scientists feel that their input is appreciated, and their opinions hold weight in decision‐making processes. This has been evidenced by instances where specific plans were modified due to the Board's intervention. The impact has been significant and direct (ESABCC expert 1). The ambitious targets set for 2040 serve as an example of the Board's influence. One scientist acknowledged that their advice to the Commission was quite shocking, as the numbers presented were not something the Commission would have proposed on its own. The scientist emphasized the necessity for Europe to take leadership in achieving the Paris targets, which requires reducing emissions by at least 90% by 2040. This case exemplifies the valuable role played by the Board (ESABCC 1).

However, there is a trade‐off between timely provision of knowledge and ensuring its quality and robustness. While acknowledging the importance of time, the Board members prioritize delivering well‐supported and actionable information that is understandable to policymakers. Therefore, they choose to spend more time gathering relevant evidence, structuring it, and ensuring its durability beyond a few weeks, with the aim of aligning with the post‐2030 policy framework (ESABCC 3).

One expert from the ESABCC expressed a sense of limited impact, primarily due to the timing their advice. If their recommendations had been made available earlier, they could have had a more significant influence on the policymaking process. However, by the time their advice was provided, most policies had already been formulated (ESABCC 1).

Regarding the question of whether the science advice given aims to be policy prescriptive, the interviews also yielded interesting insights. The IPCC, famously, aims to be policy‐relevant but never policy‐prescriptive. Reviewing the first reports and the nature of the Board's institutional set‐up, begs the question how its members interpret this aspect of knowledge provision. During the interviews, contrary to my initial expectations, it became clear that it does not aim to be policy prescriptive. “We do not try to be policy prescriptive… We do speak to the potential effectiveness [of the recommendations]. But even if you take that into account, it's not prescriptive” (ESABCC 1).

However, the picture was nuanced by one respondent, who indicated that “maybe we venture a bit further than the IPCC”. Also, because we have a clear policy framework that we can relate to. The IPCC speaks for all the nations of the world and therefore it would be difficult to pinpoint very specifically particular policies or policy frameworks. That EU policy framework, that is a consistent one where you do have a well‐defined scope of policymaking there. It makes sense and is possible then to enter that policy framework, that bubble and look around and try to be a bit more specific on what we think of those policies and which we consider a good step forward but not sufficient, which we consider desirable and which we consider also not desirable. It is a bit more prescriptive than the IPCC (ESABCC 3).

#### 5.3.9 Relationship with Other External Actors or Wider Climate Governance

The Board interacts with institutional stakeholders as well as civil society and wider public. It moreover interacts with IPCC and national advisory boards, engaging in a complex set of interactions and speaking to different audiences (general, technical, political, scientific) with a relatively limited amounts of resources.

##### IPCC

The respondent emphasized the importance of having board members who possess extensive knowledge of the workings of the IPCC, allowing them to contribute effectively. Their familiarity with the IPCC's work enabled them to guide the board to consider scenarios from the IPCC's latest assessment report. This ensured that the data and evidence relied upon by the board were consistent with that considered by the IPCC, supplemented by additional scenarios they themselves provided (ESABCC 3). Furthermore, the respondent highlighted the value of the IPCC and its Working Group 3 report in identifying desirable solutions and policies. Although these findings were more theoretical in nature, they were immensely useful as a resource to guide the board and evaluate European policies in the context of these theoretical solutions (ESABCC 3). When asked about any operational relationships between the Secretariat and the National Climate Council or the IPCC, the response indicated that direct interaction with the IPCC was primarily facilitated through its members. However, the Secretariat does not have a formal or technical relationship with the IPCC, as their involvement in the assessment cycle of the IPCC is limited, given the extensive nature of the process (ESABCC 3).

##### Other Climate Councils

There is an exchange between the Advisory Board and national boards, with joint meetings and invitations extended to representatives from the national boards. The British Council is cited as a pioneering example, having been involved in the field for a considerable time and gaining significant influence (ESABCC 1).

The Advisory Board seeks to maintain dialog with National Councils and has engaged with them on various occasions. In November 2022, representatives from existing climate councils were invited to a workshop in Copenhagen to discuss similarities, differences, and potential interplay between the European and national levels. Since then, further interactions on content‐related issues have taken place. The goal is to establish regular interactions that serve the work of the Advisory Board and the National Councils, providing a helpful dialog for both parties. However, the Advisory Board does not aim to formalize a network of climate councils at the European level, as there is an existing international climate councils’ network that seeks to facilitate interaction between councils. Over‐formalization may lead to resource‐intensive processes, which are not seen as desirable at this point (ESABCC 3). The Advisory Board prefers to maintain an ad hoc approach in engaging with National Councils. If there is a need to present the latest report or have discussions on various topics, meetings or webinars are set up. This approach is favored over establishing a new network. A European Environmental Advisory Councils Network already exists. Additionally, the implementation of climate regulations and governance primarily happens at the national level, where national climate councils play a crucial role (ESABCC 3).

The interaction between the Board and national climate advisory bodies also plays a supportive role in driving action at the national level. It helps these boards and councils advocate for necessary actions to be taken by their respective countries. The Advisory Board also aims to explore ways in which they can assist the national councils and provide meaning to decisions made at the European level, demonstrating their alignment with scientific evidence (ESABCC 3, EEA). The dialog and exchange of ideas between the two entities are considered highly useful (ESABCC 1). Another interviewee reflects on the fact that the European Advisory Board convenes national boards once a year, emphasizing the interconnectedness between the European and national levels where such boards exist. Furthermore, the European Advisory Board encourages member states to establish their own national boards. This nascent relationship between the EU board and national level boards is seen as key, as often EU climate policy encounters hurdles at the national level. The possibility of joint messaging from these multi‐level boards is seen as a potentially significant development for the future (EEA).

## 6 Conclusion

Giving a first account of the institutional design and internal knowledge production processes of the recently established ESABCC, this paper contributes to a better understanding of this novel body within the EU's climate governance architecture. Its inception can be seen as a significant institutional and policy innovation, not only for the EU but as part of a broader global trend in which climate science advisory bodies are proliferating to mediate the increasingly complex interface between scientific expertise and policymaking.

The ESABCC is particularly noteworthy as the first supranational SAB of its kind, established under the European Climate Law (2021). It is tasked with providing independent scientific advice and assessing the consistency of EU policies with the Union's climate objectives, including climate neutrality by 2050. This represents a deeper institutionalization of science advice at the EU level, within a policymaking system that has long claimed to be committed to evidence‐ and science‐based decision‐making. Given the EU's pre‐existing knowledge infrastructure—which includes inputs from international sources like the IPCC, as well as numerous committees, agencies, and expert bodies—this paper asks how the ESABCC fits within that architecture. Through a comparative analysis with the IPCC and the UK's CCC, the paper seeks to understand what kind of advisory body the ESABCC is, and what role it may come to play in the EU's climate governance.

The comparison is analytically valuable. The IPCC, the CCC, and the ESABCC are all key SABs informing climate policy, yet they differ substantially in terms of institutional design, mandate, composition, and function. These differences reflect the levels of governance they operate within—global (IPCC), national (CCC), and supranational (ESABCC)—and the distinct political and institutional logics underpinning their roles.

In terms of legal foundation and mandate, the IPCC was established in 1988 by the World Meteorological Organization and the United Nations Environment Programme to assess the global scientific literature on climate change. Its role is not to be policy prescriptive but to offer objective syntheses of climate science. The CCC, in contrast, was established under the UK's Climate Change Act (2008) with a statutory mandate to advise government on emissions targets and monitor policy implementation. The ESABCC, created by the European Climate Law, holds a narrower mandate than the CCC—it does not commission its own research—but is tasked with providing targeted advice and assessing the alignment of EU and member state policies with climate goals.

The ESABCC differs from the IPCC in that its advice must be considered by EU institutions. If the European Commission decides to deviate from the Board's recommendations, it must publicly justify doing so. This institutionalized link to EU decision‐making gives the ESABCC a level of embeddedness in policymaking that the IPCC lacks. At the same time, it resembles national councils like the CCC in being policy‐influential rather than purely deliberative. This is reflected in the European Commission recommending a 90% net greenhouse gas emission reduction by 2040, which is in line with the ESABCC's advice. However, unlike the CCC, the ESABCC does not have a statutory watchdog role or formal authority to demand data or action.

Structurally, the IPCC is a global network of thousands of volunteer scientists working through working groups and assessment cycles. It synthesizes vast volumes of peer‐reviewed research but operates with limited institutional autonomy. The CCC, by contrast, comprises a small, appointed expert body supported by a dedicated secretariat with a stable budget. Similarly, the ESABCC consists of 15 appointed members drawn from across the EU and coordinated through the EEA. However, concerns about independence persist due to its reliance on the EEA for funding and the fact that its members are unpaid, which may affect participation diversity and influence.

In terms of thematic focus, the IPCC covers a comprehensive global perspective, addressing physical science, impacts, adaptation, and mitigation. The CCC is more targeted, advising on UK emissions pathways and budget frameworks. The ESABCC occupies a hybrid position, tasked with assessing EU‐wide policy coherence and goal alignment in a multilevel governance setting. Its reports have thus far included assessments of EU climate targets and feedback on legislative proposals, which, while fewer in number than CCC outputs, are explicitly aimed at influencing strategic decisions at the EU level.

While the IPCC aspires to strict policy neutrality, the ESABCC operates in a more overtly normative space. Its scientific advice can be more policy prescriptive, especially as it is asked to assess pathways toward legally binding goals. This is reflected in its outputs, which not only summarize scientific findings but evaluate and recommend actions. Its composition, however, is limited largely to natural scientists and economists, lacking the interdisciplinary mix—including representatives of civil society—seen in some national councils. While this may enhance technical rigor and procedural efficiency, it potentially limits representativeness and inclusiveness.

The ESABCC's potential role extends beyond providing advice. It aspires to act as a convener for national climate councils across the EU, thereby reinforcing the position of scientific knowledge in national policymaking contexts. This role, however, remains aspirational and warrants further empirical investigation. In its current state, the ESABCC exhibits traits of a knowledge broker, a policy advisor, and a potential policy entrepreneur. Given its proximity to EU institutions, the degree to which it can maintain independence and avoid politicization—especially in times of political resistance to ambitious climate policy—remains a key question.

The analysis in this paper has shown that while the IPCC, CCC, and ESABCC share a commitment to informing climate policy through science, they are shaped by fundamentally different institutional arrangements. The IPCC prioritizes global scientific consensus, the CCC combines scientific advice with domestic accountability, and the ESABCC represents a novel supranational body navigating between these models. The tripartite role (advisory, watchdog and knowledge broker) can be strongly observed in the ESABCC. As EU climate governance becomes more complex and contested, the ESABCC's design and evolution will be key to understanding how scientific advice can shape and stabilize policymaking in turbulent times.

## Conflict of Interest

The authors declare no conflict of interest.

## Supporting information



Supporting Information

## Data Availability

The data that support the findings of this study are available from the corresponding author upon reasonable request.
