# Supplementary material for: The Institutional Design of Scientific Advisory Boards on Climate Change: A Comparison at the Intergovernmental, Supranational, and National Level
Source: Glob Chall. 2025 Aug 5;9(9):e00371. doi: 10.1002/gch2.202400371 (PMC12418329; doi:10.1002/gch2.202400371)
Supplement: Supplementary file 1 — Supporting Information [file GCH2-9-e00371-s001.docx]

# **Appendix**

**Data material**

**Documents:**

**ESABCC**

ESABCC (2022) Towards a climate-neutral and climate-resilient EU energy infrastructure: recommendations to ACER, available at <https://climate-advisory-board.europa.eu/reports-and-publications/towards-a-climate-neutral-and-climate-resilient-eu-energy-infrastructure-recommendations-to-acer>, accessed on 17.08.2023

ESABCC (2023a) Setting climate targets based on scientific evidence and EU values: initial recommendations to the European Commission, available at <https://climate-advisory-board.europa.eu/reports-and-publications/setting-climate-targets-based-on-scientific-evidence-and-eu-values-initial-recommendations-to-the-european-commission>, accessed on 17.08.2023

ESABCC (2023b) Addressing the energy crisis while delivering on EU’s climate objectives: recommendations to policy makers, available at <https://climate-advisory-board.europa.eu/reports-and-publications/addressing-the-energy-crisis-while-delivering-on-eus-climate-objectives-recommendations-to-policy-makers>, accessed on 17.08.2023

ESABCC (2023c) Towards a decarbonised and climate-resilient EU energy infrastructure: recommendations on an energy system-wide cost-benefit analysis, available at <https://climate-advisory-board.europa.eu/reports-and-publications/towards-a-decarbonised-and-climate-resilient-eu-energy-infrastructure-recommendations-on-an-energy-system-wide-cost-benefit-analysis>, accessed 17.08.2023

ESABCC (2023d) Scientific advice for the determination of an EU-wide 2040 climate target and a greenhouse gas budget for 2030–2050, available at <https://climate-advisory-board.europa.eu/reports-and-publications/scientific-advice-for-the-determination-of-an-eu-wide-2040>, accessed on 17.08.2023

ESABCC (2024) Towards EU climate neutrality: progress, policy gaps and opportunities, available at <https://climate-advisory-board.europa.eu/reports-and-publications/towards-eu-climate-neutrality-progress-policy-gaps-and-opportunities>, accessed on 02.02.2024

ESABCC (2024) Towards climate neutral and resilient energy networks across Europe - advice on draft scenarios under the EU regulation on trans-European energy networks, available at <https://climate-advisory-board.europa.eu/reports-and-publications/towards-climate-neutral-and-resilient-energy-networks-across-europe-advice-on-draft-scenarios-under-the-eu-regulation-on-trans-european-energy-networks>, accessed on 30.07.2024

**European Commission**

Regulation (EU) 2021/1119 of the European Parliament and of the Council of 30 June 2021 establishing the framework for achieving climate neutrality and amending Regulations (EC) No 401/2009 and (EU) 2018/1999 (‘European Climate Law’), available at <https://eur-lex.europa.eu/legal-content/EN/TXT/?uri=CELEX%3A32021R1119&qid=1720178452217>, accessed on 30.08.2023

**European Parliament/ Council of the European Union**

Regulation of the European Parliament and of the Council establishing the Framework for achieving Climate Neutrality and amending Regulations (EC) NO 401/2009 and (EU) 2018/1999 (‘EUROPEAN CLIMATE LAW’), available at <https://eur-lex.europa.eu/legal-content/EN/TXT/?uri=CONSIL%3APE_27_2021_REV_1&qid=1720178452217>, accessed on 30.08.2023

Proposal for a Regulation of the European Parliament and of the Council establishing the framework for achieving climate neutrality and amending Regulation (EU) 2018/1999 (European Climate Law) – Letter to the Chair of the European Parliament Committee on the Environment, Public Health and Food Safety (ENVI), available at <https://eur-lex.europa.eu/legal-content/EN/TXT/?uri=CONSIL%3AST_8440_2021_INIT&qid=1720178452217>, accessed on 30.08.2023

**EEA**

EEA Press Release (2022) New European Scientific Advisory Board on Climate Change appointed, available at <https://www.eea.europa.eu/en/newsroom/news/scientific-advisory-board-climate-change>, accessed on 30.08.2023

EEA Press Release (2022) EU Climate Advisory Board chair elected, available at <https://www.eea.europa.eu/en/newsroom/news/climate-advisory-board-chair-elected>, accessed on 30.08.2023

EEA (2023) Vacancy notice for ESABCC Expert, available at <https://aa254.referrals.selectminds.com/jobs/expert-european-scientific-advisory-board-on-climate-change-48>, accessed on 03.03.2023

ClimateADAPT (2021) Call for expressions of interest: Designation of the members of the European Scientific Advisory Board on Climate Change, available at <https://climate-adapt.eea.europa.eu/en/news-archive/call-for-expressions-of-interest-designation-of-the-members-of-the-european-scientific-advisory-board-on-climate-change>, accessed on 30.08.2023

EEA (2023) Scientific Committee opinion on recruitment of scientific staff in 2022, available at <https://www.eea.europa.eu/en/about/working-practices/docs-register/sc-opinion-on-recruitment-2022_oct2023_final.pdf>, accessed on 30.08.2023

**E3G**

Eichler, T., Giannelli, E., Skillings, S., van Melkebeke, T., (2022) Putting Scientific Advice At the Heart of the EU’s Climate Transition – Moving the ESABCC from Set-Up to Delivery Planning, available at <https://www.jstor.org/stable/resrep46904?seq=1>, accessed on 23.08.2023

Giannelli, E. and van Melkebeke, T. (2022) Asset or Talking Shop? Making the most of the ESABCC, available at <https://www.e3g.org/publications/asset-or-talking-shop-making-the-most-of-the-esabcc/>, accessed on 23.08.2023

**IIASA**

Byers, E., Brutschin, E., Sferra, F., Luderer, G., Huppmann, D., Kikstra, J., Pietzcker, R., Rodrigues, R., & Riahi, K., (2023) Scenarios Processing, Vetting and Feasibility Assessment for the European Scientific Advisory Board on Climate Change, *Institute for Applied Systems Analysis and Potsdam Institute for Climate Impact Research*, available at <https://pure.iiasa.ac.at/id/eprint/18828/>, accessed on 14.08.2023

**Interviews:**

| **Interviewee** | **Date** |
| --- | --- |
| ESABCC scientific expert 1 | 05.12.2023 |
| ESABCC scientific expert 2 | 24.01.2024 |
| ESABCC secretariat member | 05.02.2024 |
| EEA expert | 15.02.2024 |

**Multimedia:**

ESABCC (2024) Towards EU climate neutrality: progress, policy gaps and opportunities, available at <https://www.youtube.com/watch?v=mx6md3N4L5E>, accessed on 29.04.2024

ESABCC (2023) Presentation of advice on the EU’s 2040 climate target, available at <https://www.youtube.com/watch?v=6s1noRu8exQ>, accessed on 29.04.2024

EUClimateAction COP 27 EU Side Events (2022) The European Scientific Advisory Board on Climate Change, available at <https://www.youtube.com/watch?v=SBikuWQLMrw>, accessed on 29.04.2024

Science for Policy Podcast (2023) Ottmar Edenhofer on giving climate advice in Europe, available at <https://www.youtube.com/watch?v=lB2h4bR3snA>, accessed on 29.04.2024

Ecologic Institute (2023) Expert Conference: Charting a path towards climate neutrality in turbulent times, available at <https://www.youtube.com/watch?v=7e5viDK2_1Y>, accessed on 29.04.2024
